# Humanized Mice as a Valuable Pre-Clinical Model for Cancer Immunotherapy Research

**DOI:** 10.3389/fonc.2021.784947

**Published:** 2021-11-18

**Authors:** Morgane M. Cogels, Redouane Rouas, Ghanem E. Ghanem, Philippe Martinive, Ahmad Awada, Dirk Van Gestel, Mohammad Krayem

**Affiliations:** ^1^ Department of Radiation Oncology, Institut Jules Bordet, Université Libre de Bruxelles, Brussels, Belgium; ^2^ Laboratory of Clinical and Experimental Oncology (LOCE), Institut Jules Bordet, Université Libre de Bruxelles, Brussels, Belgium; ^3^ Laboratory of Cellular Therapy (UTCH), Institut Jules Bordet, Université Libre de Bruxelles, Brussels, Belgium; ^4^ Department of Medical Oncology, Institut Jules Bordet, Université Libre de Bruxelles, Brussels, Belgium

**Keywords:** humanized mice, preclinical model, cancer, immunotherapy, oncoimmunology

## Abstract

Immunotherapy with checkpoint inhibitors opened new horizons in cancer treatment. Clinical trials for novel immunotherapies or unexplored combination regimens either need years of development or are simply impossible to perform like is the case in cancer patients with limited life expectancy. Thus, the need for preclinical models that rapidly and safely allow for a better understanding of underlying mechanisms, drug kinetics and toxicity leading to the selection of the best regimen to be translated into the clinic, is of high importance. Humanized mice that can bear both human immune system and human tumors, are increasingly used in recent preclinical immunotherapy studies and represent a remarkably unprecedented tool in this field. In this review, we describe, summarize, and discuss the recent advances of humanized mouse models used for cancer immunotherapy research and the challenges faced during their establishment. We also highlight the lack of preclinical studies using this model for radiotherapy-based research and argue that it can be a great asset to understand and answer many open questions around radiation therapy such as its presumed associated “abscopal effect”.

## Introduction

Interest in immunotherapy, particularly in immune checkpoint inhibitors (ICIs), has grown over the past decades but their development is slowed down by the lack of adequate pre-clinical animal models allowing valid evaluation of immunotherapies and their combinations.

Immunocompetent mouse models have been used for decades in cancer research for their ability to generate antitumor-specific immune responses. However, mostly various murine tumors have been used either xenografted or induced by diverse carcinogens, often on a transgenic background. Nevertheless, these tumors had the advantage to grow in a physiologically pertinent tumor microenvironment (TME). Thus, syngeneic murine cancer cells, which are genetically identical cells cultured *in vitro* and engrafted on murine hosts, have been used although their available repertoire is limited (1) and the model lacks cell heterogeneity that characterizes cancer (1, 2). In addition, tumors tend to grow fast in these models, offering limited time for immune response to fully develop (1). Most importantly, there are inconsistencies between murine and human immune systems both innate and adaptive at different levels including cell composition, protein expression, pathway components and genomic responses (3, 4). Altogether, due to the low translational potential of such preclinical models (1), they were used with caution (5).

Genetically engineered mouse models (GEMMs) have been developed and used in cancer research. They are engineered to express an oncogene or to lose a tumor suppressor gene, which favors tumor development, possibly in a tissue-specific fashion. This model provides the advantage of slower tumor growth, allowing for prolonged immunotherapy treatment as well as a much more complete microenvironment than the one developing with injected tumor cells. However, this model has many drawbacks such as breeding difficulties, low mutational burden (unlike human tumors), and the need for non-invasive imaging techniques to screen for tumors developing in internal organs (1). Moreover, GEMMs have limited translational potential due to their fully murine immune-tumor system (3, 6).

Recent development of humanized mouse models bearing both human immune system and human tumors opened new perspectives in terms of translational value. The model consists of highly immunocompromised mice engrafted with human immune cells, which can subsequently be engrafted with a human tumor either in the form of cell line-derived xenografts (CDXs) or patient-derived xenografts (PDXs). The latter methods are widely used in cancer research, yet although CDXs are less time-consuming, the *in vitro* culture step before engraftment may lead to a substantial loss of primary tumor features (7). PDXs, on the other hand, are fresh human tumor fragments directly engrafted onto a recipient mouse, they are difficult to establish and can lose their associated human stroma over time (8). This field is currently undergoing extensive efforts to make humanized mice model a reliable and representative tool for preclinical immuno-oncology research.

## Humanization Methods

**Figure 1:**
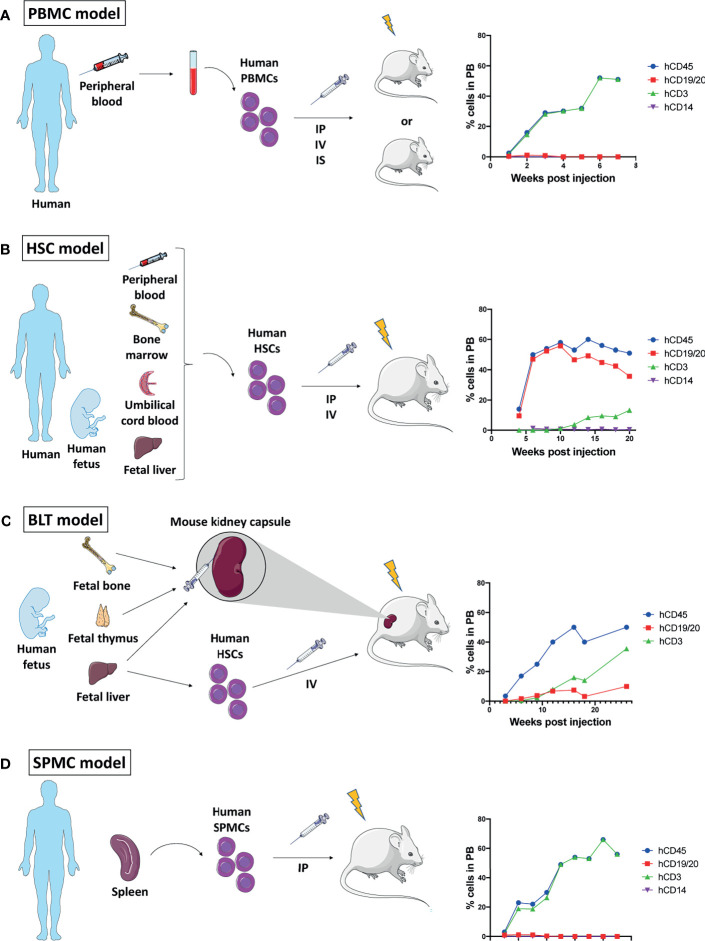
Mouse humanization models. Schematic representation of the four humanization methods described, and graphs of their immune reconstitution in peripheral blood (PB) over time showing human CD45^+^ immune cells out of all blood leukocytes and human CD19/20^+^ B cells, human CD3^+^ T cells, and human CD14^+^ monocytes/macrophages out of human CD45^+^ cells. Immune reconstitution data showed on the graphs are gathered from different studies (10–14). **(A)** Generation of peripheral blood mononuclear cells (PBMCs) humanized mouse: Human PBMCs isolated from peripheral blood are engrafted into a low dose whole body irradiated or non-irradiated immunocompromised mouse by intraperitoneal (IP), intravenous (IV) or intrasplenic (IS) administration. **(B)** Generation of hematopoietic stem cells (HSCs) humanized mouse: Human HSCs isolated from either adult peripheral blood, bone marrow or umbilical cord blood, or from fetal liver are engrafted into a low dose irradiated immunocompromised mouse by IP or IV administration. **(C)** Generation of bone, liver, thymus (BLT) humanized mouse: Human fetal bone, thymus and liver tissue are co-transplanted under the kidney capsule of an immunocompromised mouse which is then irradiated and injected IV with human HSCs isolated from fetal liver. **(D)** Generation of spleen mononuclear cell (SPMC) humanized mouse: Human SPMC isolated from an adult spleen are engrafted into a low dose irradiated immunocompromised mouse by IP administration.

The successful engraftment of human immune cells relies on a strong immunodeficiency of the recipient mouse. Mouse strains commonly used for humanization are often based on the non-obese diabetic (NOD) highly immunocompromised (SCID) background, and include NOD-SCID Gamma(null) (NSG), NOD-SCID GammaCyto(null) (NOG), and NOD Rag2(null) Gamma(null) (NRG) mice, as extensively reviewed by Martinov et al. (9). **Figure 1** summarizes the different available methods of immune cell engraftment and monitoring, and **Table 1** describes their respective advantages and disadvantages. Below, we detail and discuss each modality.

**Table 1:** Comparison of the methods used to generate humanized mice.

Method	Advantage				Disadvantage		
	HIS reconstitution	Material availability	Autologous tumor to IS	Long-term studies	GvHD	Time consuming	Technical difficulties
**PBMC**	+/−	+	+	–	+	–	–
**HSC**	+	+/−	−	+	−	+	+/−
**BLT**	++	–	–	+	+/−	+	+
**SPMC**	+/−	+/−	−	−	+	−	+/−

HIS, Human immune system; GvHD, Graft versus host disease; PBMC, Peripheral blood mononuclear cell; HSC, Hematopoietic stem cell; BLT, Bone liver thymus; SPMC, Spleen mononuclear cell; ++, Advantage or disadvantage highly present in the model; + present in the model; +/–, somewhat present in the model; –, absent from the model.

### Peripheral Blood Mononuclear Cell (PBMC) Engraftment

The administration of purified human PBMCs into immuno-compromised mice results in the partial reconstitution of a human immune system in the mouse peripheral blood and lymphoid tissue (15). Four to 12 weeks old mice can be injected by either intravenous, intraperitoneal or intrasplenic routes with 5x10^6^–20x10^6^ PBMCs (10, 16, 17). Mice can undergo low dose total body irradiation (2-2.5 Gy) before PBMC injection, but it is unclear whether this pre-conditioning provides an advantage for engraftment success (18, 19). Three to 4 weeks post-PBMC administration, high levels of human immune cells can be detected in the mouse peripheral blood (10, 15). In this model, most of the engrafted cells are human CD3^+^ T cells, and their survival in the murine host is further sustained by human PBMC cytokines, such as interleukins (IL-1β, IL-2, IL-5 and IL-10), granulocyte-macrophage colony-stimulating factor (GM-CSF), and gamma-interferon (IFNγ) (11). Yet, the lack of other human cytokines may explain the low level of human B cells and myeloid cells (20) (**Figure 1A**).

Although the quick immune engraftment renders this model attractive for research, the engrafted CD3^+^ T cells eventually react with the mouse major histocompatibility complex (MHC) class I and class II molecules to induce a xenogeneic graft versus host disease (GvHD) leading to the mouse death within 4-8 weeks (18). Despite the narrow work time window, the PBMC model is still widely used as it provides one major advantage: the possibility to engraft PBMCs and tumor tissue originating from a same patient (21).

### Hematopoietic Stem Cell (HSC) Engraftment

Hematopoietic stem cells, characterized by the expression of the cell surface marker CD34, can either be isolated from human umbilical cord blood (hUCB), bone marrow (22), peripheral blood (23), or fetal liver (24). Human UCB is the most used source of HSCs as their density is higher than in peripheral blood, and cord blood collection is not an invasive intervention, therefore more prone to be accepted by ethical committees. Human UCB-derived HSCs were also shown to have superior leukocyte reconstitution capabilities as compared to bone marrow-derived HSCs (25). Importantly, the CD34^+^ HSCs used for humanization showed superior engraftment efficacy and reconstitution potential when isolated from fresh cord blood as compared to cultured CD34^+^ HSCs (26).

Following myeloablation by low dose total body irradiation or busulfan treatment, 4 to 7 weeks-old mice are intravenously injected with 60 – 100 x 10^3^ human CB-derived CD34^+^ HSCs, which will home to the bone marrow (12, 17). Alternatively, intrahepatic injection of HSCs can be performed in newborn mice (27). Human CD45^+^ cells may be detected in the mouse peripheral blood on week 4, and they reach a plateau on weeks 8 to 10. The main reconstituted human population is the hCD19^+^ B cells while hCD3^+^ T cell count remains rather low until weeks 10 - 12 to reach their maximum level at weeks 18 to 20. hCD14^+^ monocytes can be found at a density of 1 – 5% of hCD45^+^ cells (12, 28). When human pro-T cells migrate to the mouse thymus, positive and negative selection allows for the establishment of a mature T cell population unable to attack murine tissue (29) (**Figure 1B**).

Importantly, the success of engraftment is variable and depends on several factors including the source of HSCs, the age, sex, and strain of the recipient mouse (20). In addition, the level of human immune chimerism was shown to influence tumor growth in these mice, and the ability of the reconstituted human immune system to infiltrate tumors depends both on tumor and HSC characteristics (30).

This model, although more time-demanding than the PBMC model, presents the advantage of a more completely reconstituted human immune system in the mouse, and the absence of GvHD.

### Human Fetal Bone, Liver, and Thymic Tissue (BLT) Engraftment

The co-transplantation of human fetal bone, liver, and thymus fragments under the kidney capsule of immunocompromised mice followed by low dose total body irradiation and intravenous injection of fetal liver-derived CD34^+^ HSCs yields a multilineage hematopoietic reconstitution that includes human T, B cells, macrophages, and dendritic cells (DCs) (13, 14, 31) (**Figure 1C**). Importantly, this model allows for proper education of human pro-T cells in a human thymic environment, hence the resulting human T cells are capable of human leukocyte antigen (HLA)-restricted antigen-specific reactions (13). Furthermore, antigen-specific antibody production and class-switching have been observed in these mice, indicating a functional T cell – B cell interaction (32).

Although the immune reconstitution in this model is ideal, it is technically challenging to develop, and the need for human fetal tissue is an important limitation due to the ethical questions it raises (33).

### Spleen Mononuclear Cell (SPMC) Engraftment

A fourth humanization model was recently proposed, in which SPMCs are engrafted into low dose irradiated mice (12). SPMCs are isolated from the spleen of organ donors, in which B and T lymphocytes, as well as activated memory T cells, are more abundant than in peripheral blood. The engraftment of SPMCs in NSG mice led to similar immune reconstitution and survival to what has been observed in PBMC engrafted NSG mice with the development of early GvHD (**Figure 1D**). The reliance of this model on an adult spleen donor makes it difficult to establish and excludes the possibility of an autologous model, as opposed to PBMC.

## Challenges Faced With Humanized Mice and Existing Solutions

Each humanization model has advantages and limitations, which can be tackled in various ways (summarized in **Table 2**).

**Table 2:** Limitations of humanized mouse models and how they can be addressed.

**Model**	Limitation	Possible solution	References
**PBMC**	GvHD	MHC I deficiency through deletion of *B2m*	(34)
**SPMC**		MHC I and MHC II dKO	(34, 35)
**PBMC** **SPMC** **BLT** **HSC**	IIR	Human cytokine or hormone transgenes Exogeneous human cytokine injections Human cytokine-encoding plasmids Human cytokine knock-in MSCs	(36–44) (45, 46) (47) (48) (49–51)
**HSC**	Engraftment failure	MSCs CD133^+^ HSCs	(52) (53, 54)
**HSC**	Inappropriate immune cells activity or maturation	Transgenic expression of human HLA in murine thymus	(30, 55)

PBMC, Peripheral blood mononuclear cell; SPMC, Spleen mononuclear cell; BLT, Bone liver thymus; HSC, Hematopoietic stem cell; GvHD, Graft versus host disease; IIR, Incomplete Immune Reconstitution; MHC, Major histocompatibility complex; B2m, beta 2 microglobulin; dKO, double knock-out; MSCs, Mesenchymal stem cells; HLA, human leukocyte antigen.

### Graft Versus Host Disease

The main pitfall of PBMC-humanized mice is the development of a xenogeneic GvHD which limits the timeframe of the study. The deletion of the beta 2 microglobulin (*B2m*) gene in NSG mice gave rise to the MHC class I deficient NSG *B2m* ^-/-^ mouse. This mouse model presents delayed GvHD onset as compared to NSG mice (34). In this sense, murine MHC class I deficient antigen-presenting cells (APCs) have reduced ability to stimulate human CD8^+^ T cell proliferation *in vitro* (41). Similarly, MHC class II deficiency reduces the ability of murine APCs to stimulate CD4^+^ T cell proliferation *in vitro* (56), yet the sole deficiency of MHC class II is not sufficient to confer resistance to GvHD, as opposed to MHC class I deficiency (18). Therefore, MHC class I and class II double knock-out (dKO) mice have been designed, in which human immune cells are unable to recognize and thus attack murine tissue while remaining functional (34, 35).

However, GvHD is also a concern in the SPMC humanization model and the engraftment of SPMCs in NSG-dKO mice allows to significantly prolong their survival. But the engraftment of SPMCs in NSG-dKO mice is slower than in NSG mice with a delayed expansion of hCD3^+^ T cells and with higher levels of hCD19^+^ B cells (12).

Finally, T cells develop in a human thymic environment in BLT-humanized mice, therefore they are not selected to be tolerant to murine tissue and they were shown to develop GvHD, limiting their use in long-term studies (57).

### Incomplete Immune Reconstitution

A common limitation to both PBMC and HSC models is the incomplete reconstitution of all human immune populations and different proportions of these immune cells as compared to those in humans. The cytokine environment present in mice does not support the full development of all immune lineages. Indeed, PBMC humanized mice lack proper myeloid engraftment, and have short-term natural killer (NK) and NK T cell survival (58). HSC humanized mice have high human B cell levels, and reasonable T cell levels, as well as some myeloid cells but few NK cells in the peripheral blood (12, 28).

The low myeloid cell engraftment and their decrease over time in PBMC-humanized mice can be due to the lack of human cytokines. Studies showed that the delivery of exogenous GM-CSF or Flt3-L into HSC-humanized mice led to the survival and proliferation of myeloid cells (45, 46); and the injection of human GM-CSF- and IL-4-encoding plasmids enhanced the development of DCs; while the expression of human M-CSF increased monocytes and macrophages development (47). Similarly, the NSG-SGM3 mouse strain was designed to express human *IL-3*, *GM-CSF*, and *SCF* transgenes, which allow for the development of myeloid cells upon human CD34^+^ cell engraftment (36). The hNOG EXL model, in which human *GM-CSF* and *IL-3* are also transgenically expressed, engrafts human HSCs more efficiently as compared to NSG-SGM3, and shows high development of mast cells and granulocytes (37). Models based on the 129xBalb/c background such as the MISTRG and MITRG models were also developed, respectively with and without a human *signal regulatory protein alpha* (*Sirpα*) transgene. In these models, human *M-CSF*, *GM-CSF*, *IL-3* and *thrombopoietin* (*TPO*) transgenes are expressed, enabling efficient engraftment of HSCs as well as the development of human T and B lymphocytes, NK and myeloid cells (38).

The impaired human NK cell expansion is likely due to a shortage in human IL-2 and IL-15 which are essential for human NK cell survival, activity and proliferation (59). Accordingly, in HSC-humanized mice, both the administration of exogenous human IL-15 and *Flt3/Flk2-L* encoding plasmids and the generation of human IL-15 and IL-17 double knock-in mice were shown to support NK cell development and activity (47, 48), and this was also the case in PBMC-humanized mice transgenic for human IL-15 (39). Likewise, NOG mice transgenic for human IL-2 were also shown to produce mature NK cells (40).

The effect of IL-6 was also investigated as the NOG IL-6 and the MISTRG-6 mouse models were created, both expressing human IL-6, and engrafted with human HSCs. They respectively improved immunosuppressive myeloid cell development in the context of cancer, and B cell class switching with antigen-specific IgG production (41, 42).

Mature T cell expansion in HSC humanized NSG mice was shown to be increased with the administration of IL-7 (60). However, mouse strains KO for the IL-2 receptor common gamma chain (IL2rγ) lack proper lymph nodes, which impairs the development of a functional human adaptive immune response (61). Therefore, the NOG-pRORγt-γc/GM3 transgenic mouse was created, which expresses murine IL2rγ under the lymphoid tissue inducer-specific promotor of retinoic acid receptor-related orphan receptor gamma t (RORγt), together with the expression of transgenes for human IL-2 and GM-CSF. This mouse model was able to develop lymph nodes and exhibited high levels of human T lymphocytes, antigen-specific IgG responses as well as increased IL-21-producing CD4^+^ T cells in the lymph nodes following human HSC engraftment (43). Another approach to overcome the lack of lymph nodes consists in transgenically overexpressing murine thymic stromal lymphopoietin (TSLP). Thus, a humanized Balb/c Rag2^-/-^IL2rγ^-/-^Sirpa^NOD^ TSLP (BRGST) mouse was created, developing lymph nodes with compartmentalized human B and T cells as well as a larger thymus as compared to BRGS mice, and more abundant mature B cells and IL-21 producing follicular helper T cells, all enabling adaptive immune responses (44).

The use of mesenchymal stem cells (MSCs) to improve HSC engraftment and differentiation has been investigated by multiple groups. MSCs have been shown to enhance HSC homing to murine bone marrow (52) and to improve differentiation of myeloid cells and B cells (49), as well as T cells (50) while promoting DC viability (51). However no consensus has been reached as recent research showed that human MSCs do not affect anti-tumoral immune response in PBMC or HSC humanized mice (62) and are even immunosuppressive (63).

### Inappropriate Immune Cell Activity or Maturation

Beyond the expansion of different immune cells, a humanization model must reflect the adaptive immune responses observed in humans. Yet, it was shown that HSC-humanized mice harbor T cells with little proliferative potential and high tolerance also called anergy together with a large expression of inhibitory receptors giving rise to exhausted phenotypes (30), as well as not fully mature B cells, which is likely due to the absence of a human thymic environment (64). The BLT model was proposed to overcome these issues, but it is not easy to establish.

Therefore, another approach has emerged, namely the transgenic expression of a common HLA allele in the mouse thymus. The engraftment of human HSCs with matching HLA allele could allow for a better thymic maturation of T lymphocytes. However, no consensus has been reached yet on the efficacy of this approach as conflicting results have been described (55, 65).

### Engraftment Failure

Concerning HSC humanization, some authors pointed out that CD34^+^ cells may not be the most potent repopulating cells, and suggested that the engraftment of CD133^+^ HSCs, which are precursors of CD34^+^ cells, might be more efficient in generating a human immune system in mice (53, 54). However, the homing capacity of CD133^+^ cells into the bone marrow of low dose irradiated mice is not efficient, requiring a direct administration to bone marrow (66).

Nevertheless, although all four humanization methods have drawbacks, many improvements have been proposed, either modifying the mouse strains or the humanization method, giving rise to more research opportunities.

## Humanized Mouse Models in Cancer Immunotherapy Research

Preclinical evaluation of immunotherapies remains a challenge for oncology researchers due to the lack of representative models. Several studies investigated the clinical relevance of humanized mice currently considered as a unique opportunity to assess immunotherapy efficacy and pharmacodynamics in a human immune-tumor context.

### Clinical Relevance of Humanized Mouse Models for Immunotherapy

#### Efficacy of Anti-Programmed Cell Death 1 (PD1)

The administration of anti-PD1 to NSCLC-bearing NSG mice humanized either with HSCs or with PBMCs caused significant tumor growth inhibition, in line with its efficacy observed in NSCLC patients (26, 67).

Likewise, anti-PD1 pembrolizumab treatment led to significant tumor growth inhibition as well as increased activity of tumor-infiltrating lymphocytes (TILs) in adrenocortical carcinoma (ACC) engrafted humanized mice. These results were supported by a phase II trial (NCT02673333) that showed a partial response to pembrolizumab in 23.1% of enrolled ACC patients, suggesting that humanized mice could efficiently model human response to treatment (68).

A prostate cancer bone metastases model was established in HSC-humanized NOG mice by injecting human prostate cancer cells into the tibia bone marrow of humanized mice. Tumor engraftment was efficient, and its development was similar to that of patients, supporting the use of this humanized mouse model for the study of prostate cancer bone metastases. Pembrolizumab treatment did not lead to an antitumoral effect in these mice, consistent with its inefficacy in prostate cancer patients (69).

A triple-negative breast cancer (TNBC) PDX-engrafted HSC-humanized NSG mouse model was designed as TNBC patients positive to programmed death-ligand 1 (PD-L1) can benefit from the anti-PD1 immune checkpoint inhibitors atezolizumab or pembrolizumab in combination with chemotherapy (70). Some mice had reduced tumor growth upon treatment with the anti-PD1 pembrolizumab or nivolumab, while no effect was observed upon anti-cytotoxic T lymphocyte antigen 4 (CTLA4) ipilimumab treatment (71).

Different tumor types engrafted in HSC-humanized NSG mice and treated with pembrolizumab, showed significant tumor growth delay of TNBC CDX as well as TNBC and NSCLC PDX similarly to what has been observed in the clinic (72). More specifically, the effect of pembrolizumab was CD8^+^ T cell-mediated, in TNBC tumor-bearing humanized mice. Importantly, both in bladder cancer and NSCLC PDX, the response to pembrolizumab was dependent on the HSC donor, consistent with the variable patient responses to anti-PD1 observed in the clinic (72).

#### Efficacy of Anti-Programmed Cell Death Ligand 1 (PD-L1)

Anti-PD-L1 durvalumab increased survival and delayed growth of subcutaneous PDX bladder cancer expressing PD-L1 engrafted into NOD/SCID mice having received an intraperitoneal injection of human lymphocytes. No effect has been observed in tumors expressing p53 (73). In line with these results, durvalumab response rate in bladder cancer (Trial #NCT01693562) was encouraging (74). However, it failed to bring any overall survival advantage (75).

#### Efficacy of Adoptive Cell Transfer (ACT)

An NSG mouse model was used for the study of adoptive cell transfer in the treatment of melanoma. In this model, melanoma cells and TILs from the same patient were transplanted into mice expressing human IL-2. TILs isolated from patients who responded to ACT were able to eradicate the tumors in the mouse model, while TILs isolated from non-responders were not capable to kill cancer cells (76), this further highlighting the translational potential of autologous humanized mice.

Similarly, an anti-tumoral effect of TILs isolated from human melanoma tumors was observed when transplanted into NOG-dKO mice bearing autologous melanoma tumors (77).

Altogether, these studies observed comparable efficacies of different immunotherapies in humanized mice like in the clinic.

### New Translational Advancements Brought by Humanized Mouse Models to Immunotherapy

#### Efficacy of Anti-PD1 Treatment

**Figure 2:**
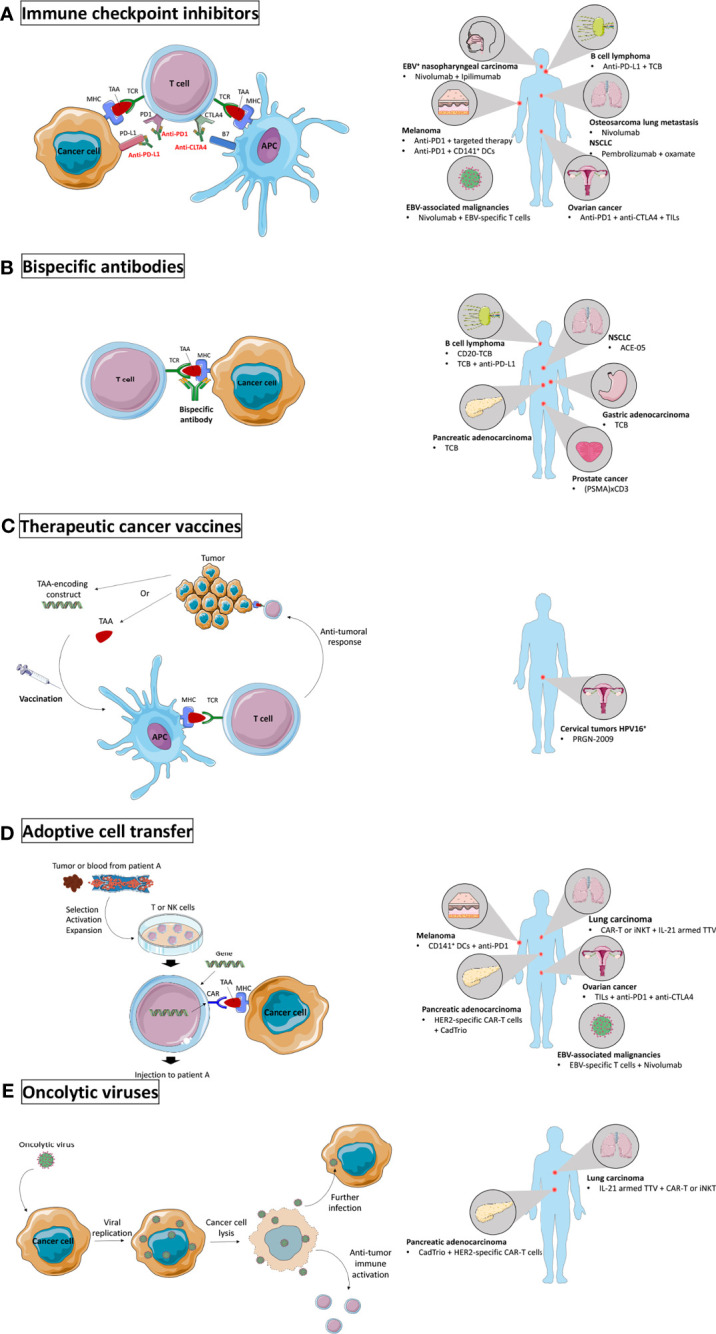
Novel immunotherapy settings tested in humanized mice. **(A)** Immune checkpoint inhibitors (ICI) prevent engagement of inhibitory receptors and allow activation of anti-tumoral immune activity. ICI were tested in humanized mice to target EBV^+^ nasopharyngeal carcinoma and other EBV-associated malignancies, melanoma, B cell lymphoma, osteosarcoma lung metastases, non-small cell lung carcinoma (NSCLC), and ovarian cancer. **(B)** Bispecific antibodies form a bridge between two target cells and were tested in humanized mice to target B cell lymphoma, pancreatic adenocarcinoma, NSCLC, gastric adenocarcinoma, and prostate cancer. **(C)** Therapeutic cancer vaccines boost the anti-tumor activity by injecting TAAs or TAA-encoding constructs to the patient. They were tested in humanized mice to target HPV16^+^ cervical tumors. **(D)** Adoptive transfer of *ex vivo* modified immune cells to improve their anti-tumor activity has been tested in humanized mice to target melanoma, pancreatic adenocarcinoma, lung carcinoma, ovarian cancer, and EBV-associated malignancies. **(E)** Oncolytic viruses specifically infect cancer cells and induce immunogenic cell death. They were tested in humanized mice to target pancreatic adenocarcinoma and lung carcinoma. Major histocompatibility complex (MHC); tumor associated antigen (TAA); T cell receptor (TCR); antigen presenting cell (APC); programmed cell death 1 (PD1); programmed death-ligand 1 (PD-L1); cytotoxic T lymphocyte antigen 4 (CTLA4); Epstein-Barr virus (EBV); cluster of differentiation (CD); dendritic cells (DCs); T cell bispecific (TCB); tumor infiltrating lymphocytes (TILs); prostate-specific membrane antigen (PSMA); human papillomavirus (HPV); natural killer (NK); chimeric antigen receptor (CAR); human epidermal growth factor receptor 2 (HER2); invariant natural killer T (iNKT); interleukin (IL); Torque Teno virus (TTV).

Although no effect of nivolumab was observed on tumor growth in PBMC-humanized mice engrafted with osteosarcoma tumors, it partially inhibited lung metastases formation by the increase of CD4^+^ and CD8^+^ T lymphocytes and of CD8^+^ T cell cytolytic activity in the lungs (78), opening the door for anti-PD1 treatment in metastatic osteosarcoma patients (**Figure 2A**).

#### Efficacy of Bispecific Antibodies

In HSC-humanized NSG mice engrafted with human B cell lymphoma, an anti-tumoral effect of the T cell bispecific antibody CD20-TCB was observed, mediated by the fast formation of stable T cell-tumor cell synapses inducing tumor cytotoxicity and cytokine synthesis, and by T cell recruitment to the tumor as well as resident T cell proliferation (79). In line with these results, TCB treatment reduced tumor growth and increased T cell infiltration in tumors in a similar B cell lymphoma humanized mouse model as well as in gastric adenocarcinoma and pancreatic adenocarcinoma (**Figure 2C**). TCB was also shown to upregulate PD1 expression on T cells and PD-L1 expression on cancer cells and T cells, and the combination of TCB with anti-PD-L1 to have a stronger anti-tumoral effect as compared to either treatment given as monotherapy (80) (**Figures 2A, C**). The use of bispecific antibodies is also supported by the efficacy of ACE-05, targeting PD-L1 and CD3, in PBMC-humanized mice bearing NSCLC tumors. ACE-05 induced tumor regression while presenting reduced off-target toxicity (81) (**Figure 2C**).

Finally, the use of bispecific antibodies was investigated in prostate cancer. Prostate-specific membrane antigen (PSMA)-CD3 T-cell recruiting bispecific antibodies were established and their use in NSG PBMC-humanized mice reduced lung metastases and eradicated large subcutaneous prostate tumors (82) (**Figure 2C**).

#### Efficacy of Therapeutic Cancer Vaccines

Human papillomavirus (HPV)-oncogenesis being associated with the constitutive expression of HPV proteins E6 and E7 in tumors, many clinical trials have investigated the efficacy of therapeutic vaccines targeting E6 and E7, but none has led to approval for clinical use so far (83). However, a novel therapeutic gorilla adenovirus HPV vaccine, PRGN-2009, has shown promise when tested in PBMC-humanized NSG mice bearing human HPV16^+^ cervical tumors as it reduced tumor growth and increased CD8^+^ and CD4^+^ T cell levels in the TME, supporting its use in clinical trials (84) (**Figure 2D**).

#### Efficacy of Combination Treatments

Epstein-Barr virus (EBV) associated nasopharyngeal carcinoma (NPC) is a highly inflamed tumor type characterized by lymphocyte infiltration and PD-L1 expression, making it an ideal candidate for immunotherapy, but representative preclinical models are lacking. Recently, an HSC-humanized NSG mouse model carrying EBV^+^ NPC PDXs was established. Treatment with nivolumab/ipilimumab combination did not result in tumor growth inhibition, yet an inflammatory response was observed demonstrated by the upregulation of pro-inflammatory cytokines and a shift in TILs composition in favor of CD8^+^ T cells (85) (**Figure 2A**). In another study using HSC-humanized NRG mice, EBV^+^ malignancies were targeted with adoptively transferred EBV-specific T cells alone or in combination with nivolumab. ACT alone showed therapeutic benefit potentiated by nivolumab, leading to reduced tumor growth and improved overall survival (86) (**Figures 2A, B**).

Ovarian cancer (OVC) patients show low response rates to single-agent immune checkpoint inhibitors (87, 88), hence clinical trials currently investigate the effects of their combination. To deepen these investigations, an NSG-HSGM3 mouse model was established, expressing both HHD II (HLA A*0201) and SGM3 transgenes and engrafted with OVC PDXs. The combination of anti-PD1, anti-CTLA4 and adoptive transfer of autologous TILs led to tumor regression, encouraging the path of combination immunotherapies in ovarian cancer (89) (**Figures 2A, B**).

The efficacy of oncolytic viruses, which selectively target cancer cells resulting in immunogenic cell death, can be improved by expressing immunoregulators. A recent study identified IL-21 to be a good candidate to improve Torque Teno oncolytic virus (TTV) efficacy in lung carcinoma tumors alone or in combination with chimeric antigen receptor T (CAR-T) or invariant natural killer T (iNKT) cell therapy in a humanized mouse model. The study showed a synergistic effect of both combination therapies, suggesting that other immunotherapies may also work synergistically with IL-21-armed TTV (90) (**Figures 2B, E**).

Pancreatic adenocarcinoma (PAC) tumors are mostly considered “cold” as very low anti-tumoral immune activation occurs and are therefore associated with poor response to immunotherapies such as ICIs. Combination therapies were therefore studied in humanized mice to tackle unresponsiveness of PAC to immunotherapy. As high expression of human epidermal growth factor receptor 2 (HER2) in PAC is associated with poor prognosis, the study showed the activity of HER2-specific CAR-T cell (HER2.CART) to improve by the addition of an oncolytic virus (CAdTrio) which produces IL-12, immune checkpoint inhibition anti-PD-L1, and safety switch HSVtk. CAdTrio intratumoral administration enhanced HER2.CART migration to the primary tumor site and stimulated the systemic immune response which led to both primary tumor eradication and metastatic disease control, providing new combination options to tackle pancreatic cancer (91) (**Figures 2B, E**).

The combination of immunotherapy with other therapies is also needed to tackle the main challenges faced by immunotherapy, i.e. the lack of immune activation and infiltration in tumors. For example, melanoma patients are widely treated with PD1 inhibitors, however 40 to 45% face primary resistance to treatment (92). A humanized mouse melanoma model, in which NSG mice humanization was realized through fetal liver-derived HSCs injection together with autologous thymus engraftment was recently described. These mice were engrafted with HLA-matched human melanoma cells, and they showed that while anti-PD1 treatment only partially reduced tumor growth, the combination of anti-PD1 with targeted therapy receptor tyrosine kinase inhibitor (TKI) sunitinib or imatinib led to complete tumor regression associated with mast cell depletion. Accordingly, tumor-infiltrating mast cells were identified as a possible cause of resistance to anti-PD1 therapy (93) (**Figure 2A**). Another combinatory approach to tackle melanoma using humanized mice consists in enhancing CD141^+^ DC number and function in addition to anti-PD1 treatment. Indeed, this subset of DCs, which is essential for CD8^+^ T cell anti-tumoral response, was shown to be decreased in advanced melanoma patients’ blood. In addition, the treatment of HSC-engrafted NSG-SGM3 mice bearing melanoma tumors with anti-PD1 combined with Flt3-L and a toll like receptor (TLR)-3 agonist resulted in a synergistic effect leading to limit the tumor growth. Similarly, the direct injection of CD141^+^ DCs intratumorally combined with anti-PD1 treatment reduced tumor growth. These results demonstrate the importance to restore CD141^+^ DC activity in advanced melanoma patients when an immunotherapeutic approach is envisaged (94) (**Figures 2A, B**).

In the search for new combination strategies, attention has been brought to glycolysis, the main energy metabolism used by cancer cells. The competition for glucose in the TME and the production of lactic acid favored by anoxia (resulting from glycolysis) play a central role in tumor cell invasion, metastases, angiogenesis, and immune hampering. The inhibition of lactate dehydrogenase subunit A (LDH-A) in glycolysis by oxamate is a promising approach in cancer treatment. To investigate the role of oxamate alone or in combination with pembrolizumab in the treatment of NSCLC, a PBMC-humanized mouse model was established. This model showed, while oxamate or pembrolizumab alone delayed tumor growth, their combination to be more efficient (95) (**Figure 2A**). This study successfully used humanized mice to demonstrate that new combination therapies can improve responsiveness to immunotherapy.

Taken together, humanized mice have widely been used as a preclinical model in cancer immunotherapy studies over the past years. They allowed to investigate therapies in new settings and new combinations (**Figure 2**) and will undoubtedly give rise to and complement clinical trials.

## Future Directions

Interest in humanized mice has grown over the past decades as it has become clearer that simple immunocompetent mice are not an optimal preclinical model, especially when studying immunotherapies and combination therapies. A preclinical model reproducing human immune-tumor interactions is thus of great interest.

In all four humanization methods and tumor-engraftment procedures described hereinabove, improvements remain to be made. Numerous studies to overcome the limitations of humanized mice are ongoing and an increasing number of humanized mouse models are being marketed. This panel of humanized mice with different properties will allow researchers to select the most appropriate model tailored for their specific application.

While more humanized mouse models are created, their applications in immuno-oncology are extending. Indeed, several aspects of immuno-oncology still remain to be studied in humanized mice, such as combination treatments with ICIs and radiotherapy for the study of the controversial “abscopal effect” specifically, following the finding that moderate hypofractionated radiotherapy rather than monofractionated or high dose hypofractionated radiotherapy is determining for anti-tumoral immune activation (96). Hence, multiple clinical trials were launched on the combination of ICIs and fractionated radiotherapy intending to enhance both a local and a systemic anti-tumoral immune response. Such clinical trials were performed in NSCLC (97) and in head and neck squamous cell carcinoma (98), where they showed great promise as the treatment was reported to be safe, well-tolerated and to induce a major response in 53.3% and 86% of patients under the combination in each tumor respectively. Complementing these clinical trials with preclinical humanized mice studies would lead to a better understanding of the underlying molecular mechanisms and could help select optimal treatment doses and schedule, which would significantly accelerate the research processes leading to faster translation strategies.

Altogether, humanized mice represent a promising preclinical model in the field of immuno-oncology and their use will not only accelerate the development of new efficient and safe cancer immunotherapies but will also drive personalized medicine into the clinic and is likely to tremendously increase our understanding of important cancer mechanisms.

## Author Contributions

MC collected the data and drafted the manuscript. MK revised the manuscript. DG, GG, PM, RR, and AA provided additional revisions. All authors contributed to the article and approved the submitted version.

## Funding

This work has been supported by “Les Amis de l’Institut Jules Bordet”.

## Conflict of Interest

The authors declare that the research was conducted in the absence of any commercial or financial relationships that could be construed as a potential conflict of interest.

## Publisher’s Note

All claims expressed in this article are solely those of the authors and do not necessarily represent those of their affiliated organizations, or those of the publisher, the editors and the reviewers. Any product that may be evaluated in this article, or claim that may be made by its manufacturer, is not guaranteed or endorsed by the publisher.

## Glossary

**Table:**

ACC	Adrenocortical carcinoma
ACT	Adoptive cell transfer
APC	Antigen presenting cell
B2m	Beta 2 microglobulin
BLT	Bone
liver	and thymus
BRGS	Balb/c Rag2^-/-^IL2rγ^-/-^Sirpa^NOD^
BRGST	Balb/c Rag2^-/-^IL2rγ^-/-^Sirpa^NOD^ Thymic stromal lymphopoietin
CAR-T	Chimeric antigen receptor T
CD	Cluster of differentiation
CDX	Cell line-derived xenograft
CTLA4	Cytotoxic T lymphocyte antigen 4
DC	Dendritic cell
dKO	Double knock-out
EBV	Epstein-Barr virus
Fab	Antigen binding fragment
Flt3-L	FMS-like tyrosine kinase 3 ligand
FoxN1	Forkhead box protein N1
GEMM	Genetically engineered mouse model
GM-CSF	Granulocyte-macrophage colony-stimulating factor
GvHD	Graft versus host disease
HER2	Human epidermal growth factor receptor 2
HIS	Human immune system
HLA	Human leukocyte antigen
HPV	Human papillomavirus
HSC	Hematopoietic stem cell
hUCB	Human umbilical cord blood
IP	Intraperitoneal
IS	Intrasplenic
IV	Intravenous
ICI	Immune checkpoint inhibitor
IFNγ	Gamma Interferon
IL	Interleukin
IL2rγ	IL-2 receptor common gamma chain
iNKT	Invariant natural killer T
Jak	Janus kinase
KO	Knock-out
LDH	Lactate dehydrogenase
MHC	Major histocompatibility complex
MSC	Mesenchymal stem cell
NK	Natural killer
NOD	Non-obese diabetic
NOG	NOD-SCID GammaCyto(null)
NPC	Nasopharyngeal carcinoma
NRG	NOD Rag2(null) Gamma(null)
NSG	NOD-SCID Gamma(null)
OVC	Ovarian cancer
PAC	Pancreatic adenocarcinoma
PBMC	Peripheral blood mononuclear cell
PD-L1	Programmed death-ligand 1
PD1	Programmed cell death 1
PDX	Patient-derived xenograft
PSMA	Prostate-specific membrane antigen
RORγt	retinoic acid receptor-related orphan receptor gamma t
SCF	Stem cell factor
SCID	Severe combined immunodeficient
Sirpα	signal regulatory protein alpha
SPMC	Spleen mononuclear cells
TAA	Tumor associated antigen
TCB	T cell bispecific
TCR	T-cell receptor
TIL	Tumor-infiltrating lymphocyte
TKI	Receptor tyrosine kinase inhibitor
TLR	Toll like receptor
TME	Tumor microenvironment
TNBC	Triple-negative breast cancer
TPO	Thrombopoietin
Treg	Regulatory T cell
TSLP	thymic stromal lymphopoietin
TTV	Torque Teno Virus
